# Rhinocerebral mucormycosis secondary to severe acute pancreatitis and diabetic ketoacidosis: a case report

**DOI:** 10.1186/s13000-021-01094-3

**Published:** 2021-04-21

**Authors:** Jinjing Wang, Yao Li, Shuai Luo, Hong Zheng

**Affiliations:** Department of Pathology, The Affiliated Hospital of Zunyi Medical University, Zunyi City, Guizhou Province P.R. China

**Keywords:** Rhinocerebral mucormycosis, Severe acute pancreatitis, Diagnosis, Pathology, Case report

## Abstract

**Introduction:**

Rhinocerebral mucormycosis is a rare and severe form of opportunistic fungal infection that can develop rapidly and cause significant mortality, particularly among diabetic patients suffering from ketoacidosis. Diagnosing rhinocerebral mucormycosis during the early stages of infection is challenging.

**Case presentation:**

We describe a case of rhinocerebral mucormycosis secondary to severe acute pancreatitis in a patient suffering from diabetic ketoacidosis. In this case, the condition was not diagnosed during the optimal treatment window. we therefore provide a thorough overview of related clinical findings and histopathological characteristics, and we discuss potential differential diagnoses.

**Conclusions:**

In summary, we described a case of rhinocerebral mucormycosis secondary to severe acute pancreatitis in a patient suffering from diabetic ketoacidosis, with the optimal treatment window for this condition having been missed. This report suggests that a definitive mucormycosis diagnosis can be made based upon tissue biopsy that reveals the presence of characteristic hyphae. Early diagnosis and treatment are essential in order to improve patient prognosis.

## Introduction

Rhinocerebral mucormycosis is a rare and severe form of opportunistic fungal infection that can develop rapidly and cause significant mortality, particularly among diabetic patients suffering from ketoacidosis [1, 2]. As the initial clinical symptoms of mucormycosis can be nonspecific and inconsistent, diagnosis is often delayed and rates of misdiagnosis are high such that the condition is not often treated effectively during the optimal treatment window [3]. The development of novel approaches to diagnosing and treating this condition during its early stages is thus essential in order to minimize patient morbidity and mortality [3].

Herein, we describe a case of rhinocerebral mucormycosis secondary to severe acute pancreatitis in a patient suffering from diabetic ketoacidosis. In this case, the condition was not diagnosed during the optimal treatment window. To improve current understanding of this condition and to better improve future diagnostic accuracy, we therefore provide a thorough overview of related clinical findings and histopathological characteristics, and we discuss potential differential diagnoses.

## Case presentation

**Fig. 1:**
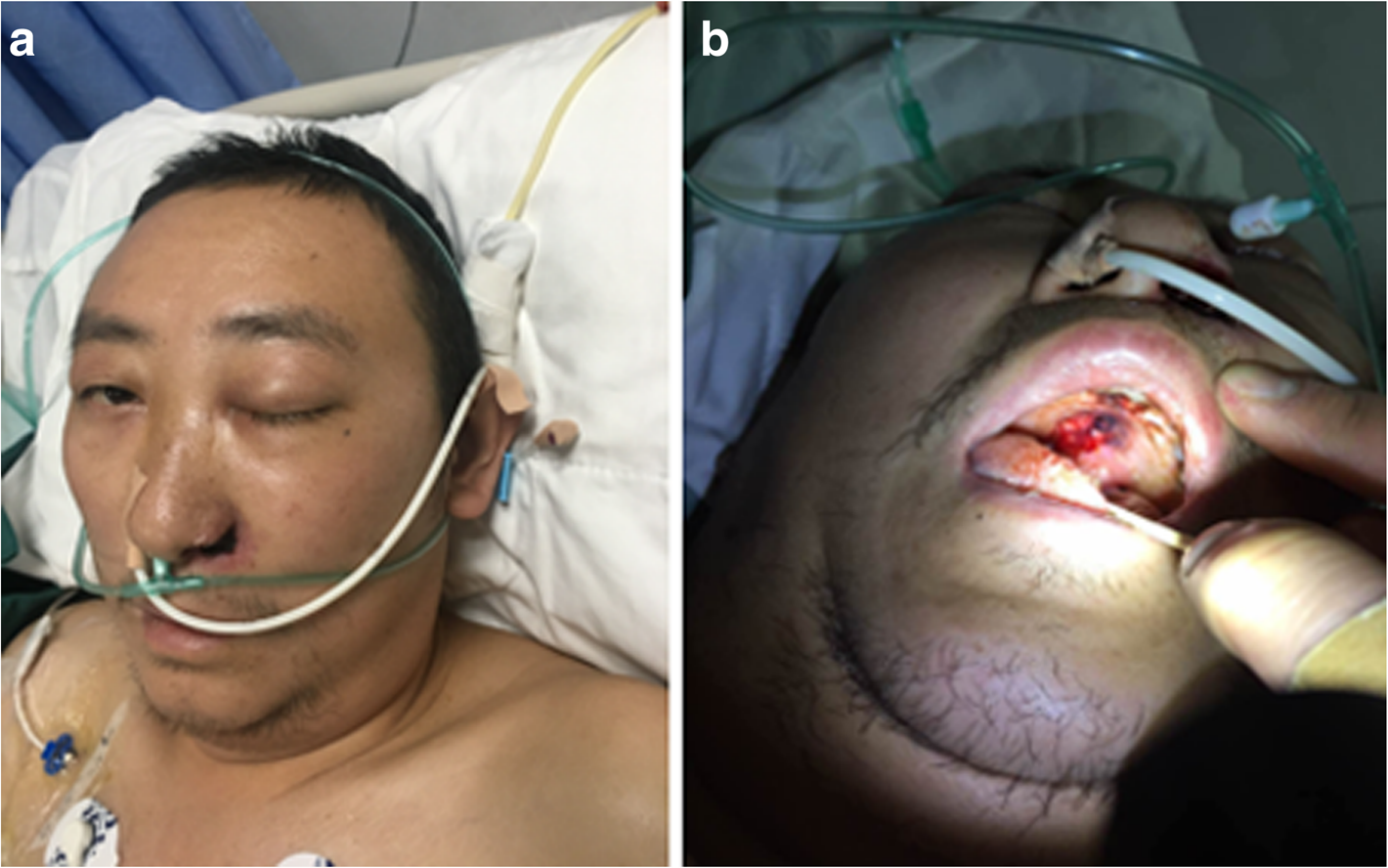
**a** Necrosis of the left nasal wing, welling of the left eye, and right side paralysis were detected. **b** Hard palate perforation

**Fig. 2:**
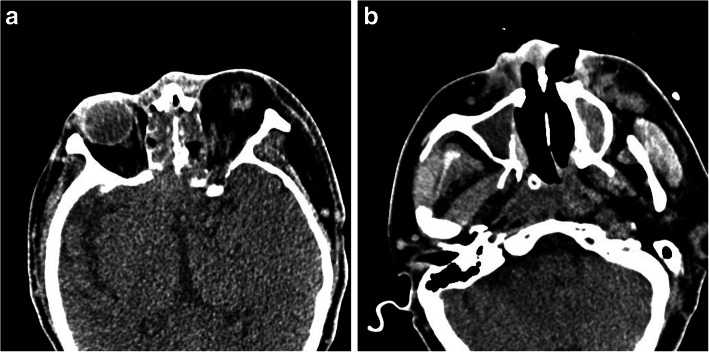
**a** Computed tomography (CT) scanning of the brain and orbits revealed extensive involvement of the bilateral intranasal sinuses, orbits, extraocular muscles, and soft tissues. Increased soft tissue density was evident in the bilateral maxillary sinus, ethmoid sinus, sphenoid sinus, and around the orbits. Dilatation of the extraocular muscles was also observed, as was erosion of the left lateral wall of the sphenoid and cribriform plate. **b** Perforation of the patient’s nasal septum affected the left nasal cavity, with evidence of hard palate and left nasal wing involvement and perforation

A 38 years old male patient with uncontrolled diabetes was admitted to our hospital complaining of severe abdominal pain after meals with nausea and vomiting. Laboratory analyses revealed high (+++) urinary ketone body levels, elevated (+) levels of urine glucose, and 24 h blood glucose fluctuations from 16.0–21.6 mmol/L. Abdominal enhanced computed tomography (CT) imaging revealed evidence of acute pancreatitis and peritoneal fluid accumulation. The patient was therefore diagnosed with acute pancreatitis, hyperlipidemia, and diabetic ketoacidosis. However, 5 days later, dark necrotic tissue was observed in the left nasal cavity of this patient. At this same time point, the tissue on the left side of his face was visibly swollen, with left orbital proptosis, eyelid drooping, complete ophthalmoplegia, and dilation of his right pupil with a marked reduction in visual acuity (Fig. 1). CT analyses revealed an increase in soft tissue density in the patient’s bilateral maxillary sinus, ethmoid sinus, sphenoid sinus, and right frontal sinusitis. Dilatation of his left medial and inferior recti was observed, and the perforation of the patient’s nasal septum was observed, affecting his left nasal cavity. Hard palate perforation was also observed (Fig. 2). Nasoendoscopy revealed large quantities of dark necrotic tissue within the bilateral nasal cavity. The majority of the soft tissue within the nasal cavity was damaged, with complete exposure of the bones of the nasal floor and the lower nasal passage, and with complete exposure of the lower nasal passage. The top of the nasal cavity was also affected. A sample of the observed dark necrotic soft tissue was excised for pathological examination.

**Fig. 3:**
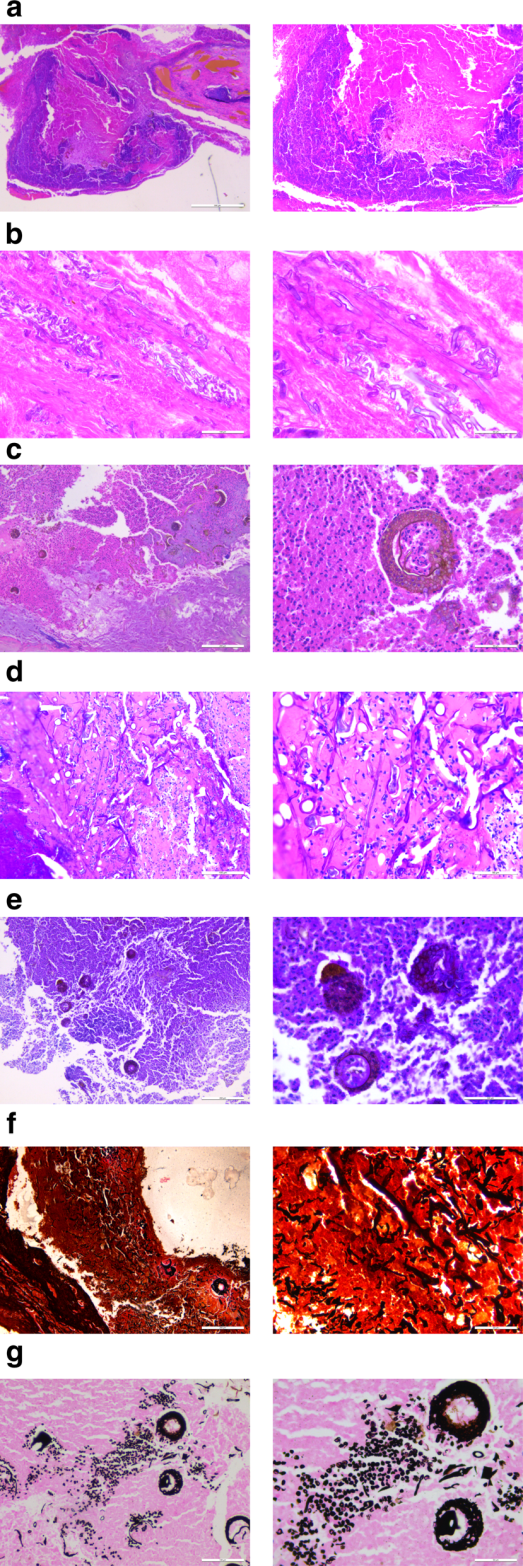
**a** Coagulated necrotic soft tissue was assessed via microscopy, revealing the presence of lymphocytes, plasma cells, and multinucleated giant cells in the necrotic tissue (A1 H&E stain × 50; A2 H&E stain × 100). **b** Many hyphae were detected in necrotic soft tissue samples, surrounding and invading the walls of blood vessels, thereby resulting in vasculitis and thrombus formation (B1 H&E stain × 200; B2 H&E stain × 400). **c** Mucorales were characterized by small round brown spores and basophilic chrysanthemum-like sporangium exhibiting sporangiospores and sporangiophores (C1 H&E stain × 100; C2 H&E stain × 400). **d**-**g** Periodic-acid-Schiff (PAS) staining revealed fungal hyphae that were pink (D1 PAS stain × 200; D2 PAS stain × 400), spores that were yellow-brown (E1 PAS stain × 200; E2 PAS stain × 400). Grocott staining highlighted hyphae (F1 Grocott stain × 100; F2 Grocott stain × 400), sporangia, and spores that were noticeably dark brown (G1 Grocott stain × 100; G2 Grocott stain × 400). H&E: Hematoxylin and eosin

Histopathological examination revealed that the coagulated necrotic tissue contained lymphocytes, plasma cells, and multinucleated giant cells (Fig. 3A1, 2). Many hyphae were visible surrounding and invading blood vessel walls, resulting in vasculitis and thrombus formation (Fig. 3B1, 2). Broad tubular hyphae that were 20–30 μm wide were observed, with two asymmetric sidewalls that were partially swollen and distorted without separation, aside from a few disorderly branches occurring at right angles (Fig. 3B2). Sporangia were characterized by small round brown spores and basophilia chrysanthemum-cluster-like sporangium, with visible endospores in the sac. Individual spores were roughly 10 μm in diameter, and were densely arranged in sheets (Fig. 3C1, 2). Periodic-acid-Schiff (PAS) staining revealed pink fungal hyphae (Fig. 3D1–2), and yellow-brown spores (Fig. 3E1, 2). Grocott staining highlighted hyphae (Fig. 3F1, 2), sporangia, and spores, which were a noticeable dark brown color (Fig. 3G1, 2). Such fungal morphology was suggestive of a Mucor fungus, and the patient was pathologically diagnosed with acute invasive mucormycosis. He was treated via surgical debridement combined with intravenous amphotericin B administration. However, due to his advanced disease progression, the patient died 4 weeks following admission.

## Discussion and conclusions

Rhinocerebral mucormycosis is a rare, aggressive, and life-threatening fungal infection [4], occurring most often in diabetes patients suffering from ketoacidosis [5]. At present, owing to the nonspecific early-stage symptoms of this disease, it is difficult to diagnose until it is relatively advanced.

Ocular manifestations are often the first presenting symptoms in patients suffering from rhinocerebral mucormycosis. These symptoms can include prominent eyeballs, swelling and redness around the eyes, impaired eye movement, decreased vision, and potentially blindness. Nasal black eschar formation is one of the most important clinical findings associated with rhinocerebral mucormycosis [4]. CT or magnetic resonance imaging (MRI) examinations of the skull generally reveal the thickening of the sinus mucosa, sinusitis, bone destruction, and cavernous sinus thrombosis. These findings may be accompanied by facial swelling and numbness, local facial skin ulceration, local black necrotic eschar formation, and black eschar or ulcers on the epiphysis.

There are several approaches that can be used to diagnose rhinocerebral mucormycosis. Fungal culture is highly specific, but the sensitivity of this approach is just 25%, limiting its clinical applicability [6]. Pathological assessment of biopsy samples can achieve a more definitive diagnosis, as affected patients generally exhibit large areas of coagulative necrotic tissue, fungal granuloma, fungal vasculitis, thrombosis, and bone destruction [5, 6]. Typical granulomatous tissues from affected patients generally contain hyphae and neutrophils surrounded by epithelioid cells, multinucleated giant cells, varying numbers of plasma cells, lymphocytes, and eosinophils. Perivascular and blood vessel invasion by fungal hyphae results in arterial thrombosis and subsequent necrosis. Hyphae are generally broad and irregularly shaped, with branches primarily forming at right angles [6]. Hematoxylin and eosin staining of these hyphae largely fails to differentiate them from background tissues, and high magnification is often necessary to clearly resolve these fungal structures. PAS and Gomori methenamine silver (GMS) staining result in the purple-red and brown-black coloration to these hyphae, respectively, making them easier to observe [4, 6]. Molecular biology approaches can also be used to diagnose this condition, although it remains challenging to leverage a broad-spectrum for the diagnosis of mucormycosis in clinical settings [6].

From a differential diagnosis perspective, rhinocerebral mucormycosis may be confused with other forms of fungal sinusitis. Identification is mainly based on the morphological characteristics of fungal hyphae. Mucor mycelia are relatively thick (6–25 μm in diameter) and disordered, with thick walls, asymmetry, little separation, partial swelling and twisting, and relatively few branches that are primarily at right angles [5, 6]. In contrast, Candida mycelia are thin (2 - 4 μm in diameter) and can be bead-like [6], while Aspergillus mycelia are of medium thickness (5–10 μm in diameter), uniform in thickness, often exhibit directional growth, are frequently separated, and generally exhibit many branches that are most often formed at acute angles [3].

Treatment options for rhinocerebral mucormycosis include the active treatment of primary disease, correction of underlying acidosis, and the early administration of agents including amphotericin B liposomes, together with the thorough debridement of local necrotic tissue [6]. Surgical and antifungal treatments generally form the cornerstones of treatment [4], and delays in initiating amphotericin B treatment for more than 6 days is associated with a doubling of mortality at 12 weeks.

In summary, we described a case of rhinocerebral mucormycosis secondary to severe acute pancreatitis in a patient suffering from diabetic ketoacidosis, with the optimal treatment window for this condition having been missed. This report suggests that a definitive mucormycosis diagnosis can be made based upon tissue biopsy that reveals the presence of characteristic hyphae. Early diagnosis and treatment are essential in order to improve patient prognosis.

## Data Availability

All the data regarding the findings are available within the manuscript.

## Abbreviations

CT: Computed tomography
MRI: Magnetic resonance imaging
PAS: Periodic-acid-Schiff
GMS: Gomori methenamine silver
